# Arbitrary shaped beam scattering from a chiral-coated conducting object with arbitrary monochromatic illumination

**DOI:** 10.1038/s41598-018-30596-2

**Published:** 2018-08-17

**Authors:** Mingjun Wang, Huayong Zhang, Xizheng Ke, Guosheng Liu, Xiaoping Ouyang

**Affiliations:** 1 School of Automation and Information Engineering, Xi’an University of Technology, Xi’an, Shaanxi 710048 P. R. China; 2 School of Electronics and Information Engineering, Anhui University, Hefei, Anhui 230039 P. R. China; 3 Department of Earth, Ocean and Atmospheric Science, Florida State University, Tallahassee, Florida 32306-4520 USA

## Abstract

An exact semi-analytical method of calculating the scattered fields from a chiral-coated conducting object under arbitrary shaped beam illumination is developed. The scattered fields and the fields within the chiral coating are expanded in terms of appropriate spherical vector wave functions. The unknown expansion coefficients are determined by solving an infinite system of linear equations derived using the method of moments technique and the boundary conditions. For incidence of a Gaussian beam, circularly polarized wave, zero-order Bessel beam and Hertzian electric dipole radiation on a chiral-coated conducting spheroid and a chiral-coated conducting circular cylinder of finite length, the normalized differential scattering cross sections are evaluated and discussed briefly.

## Introduction

The electromagnetic (EM) properties of chiral media have been extensively investigated in past several decades, for their wide applications in so many fields^1–5^. Undoubtedly, EM scattering is a canonical problem for analyzing the interaction of EM waves with chiral media. Utilizing the vector wave functions (VWFs), Bohren examined the plane wave scattering from an optically active sphere or cylinder^6,7^. The extended boundary condition method (EBCM) or T-matrix method has been effectively applied to the scattering by a chiral object or aggregated optically active particles^8–10^. The method of moments (MoM) with surface formulations has been presented by Worasawate *et al*.^11^, and the bi-isotropic finite difference time domain technique by Semichaevsky *et al*.^12^, for treating the plane wave scattering by a chiral object. Dmitrenko *et al*. proposed a numerical method of discrete sources to calculate the scattered fields by a conducting body with a homogeneous chiral coating^13^. Recently, we have developed an approach for computing arbitrary shaped beam scattering from a chiral object by combining the field expansions in terms of the spherical VWFs and the MoM scheme^14^. However, the EM process in a chiral medium coated on a conducting object is often of great importance in our research on antenna radomes, microstrip substrates and waveguides. In this paper, based on our previous work a semi-analytical solution is introduced on the scattering from a chiral-coated conducting object.

We provide the theoretical analysis in section 2 for the determination of the scattered fields of an EM beam from a chiral-coated conducting object. In section 3, the far-field scattering cross sections are computed for a Gaussian beam, circularly polarized wave (CPW), zero order Bessel beam (ZOBB) and Hertzian electric dipole (HED) radiated field striking a chiral-coated conducting spheroid and finite-length circular cylinder. The conclusion is in Section 4.

## Formulation

**Figure 1:**
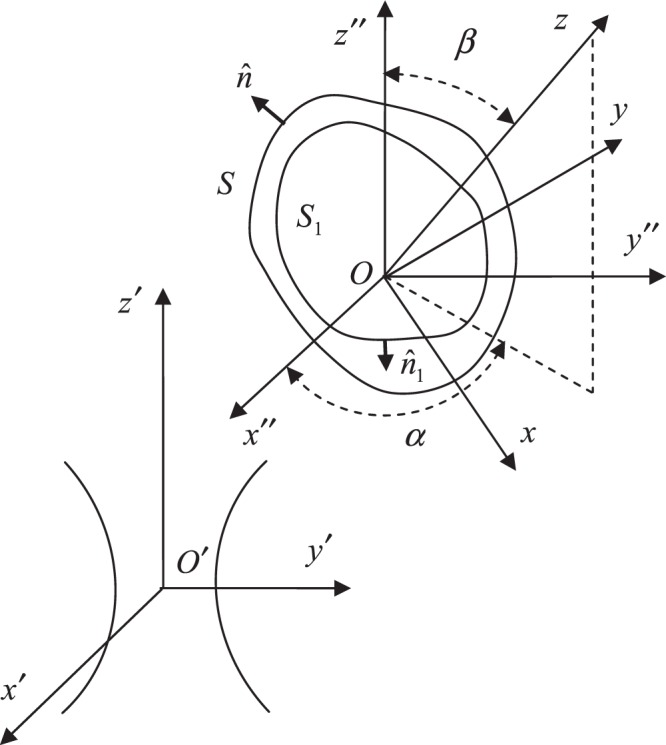
Geometry for the scattering problem.

As shown in Fig. 1, an EM beam is propagated along the positive *z*′ axis in *O*′*x*′*y*′*z*′. The system *Ox*″*y*″*z*″ is parallel with *O*′*x*′*y*′*z*′, the point *O* is at (*x*_0_, *y*_0_, *z*_0_) in the Cartesian coordinate system *O*′*x*′*y*′*z*′. A conducting object having a chiral coating is attached to the system *Oxyz*, which is rotated with respect to *Ox*″*y*″*z*″ through Euler angles *α* and *β*^15^. An exp(−*iωt*) time-harmonic convention is assumed for the EM fields in this paper.

Since the scattered wave behaves as a spherical diverging wave emanating from the center of the scatterer, the scattered fields from the chiral-coated conducing object can be expanded in an infinite series of spherical VWFs with regard to in coordinate system *Oxyz*, as following^14^:

1 $${{\bf{E}}}^{s}={E}_{0}\,\sum _{m=-\infty }^{\infty }\,\sum _{n=|m|}^{\infty }\,[{\alpha }_{mn}{{\bf{M}}}_{mn}^{(3)}({k}_{0})+{\beta }_{mn}{{\bf{N}}}_{mn}^{(3)}({k}_{0})]$$

2 $${{\bf{H}}}^{s}=-\,i{E}_{0}\frac{1}{{\eta }_{0}}\,\sum _{m=-\infty }^{\infty }\,\sum _{n=|m|}^{\infty }\,[{\alpha }_{mn}{{\bf{N}}}_{mn}^{(3)}({k}_{0})+{\beta }_{mn}{{\bf{M}}}_{mn}^{(3)}({k}_{0})]$$

where ${k}_{0}=\omega \sqrt{\mu {}_{0}\varepsilon _{0}}$, ${\eta }_{0}=\sqrt{{\mu }_{0}/{\varepsilon }_{0}}$ are respectively the wavenumber of the incident wave and the characteristic impedance of free space, and *α*_*mn*_, *β*_*mn*_ are the unknown expansion coefficients to be determined.

A chiral medium can be characterized by the following constitutive relations^16^

3 $${\bf{D}}={\varepsilon }_{0}{\varepsilon }_{r}{\bf{E}}+i\kappa \sqrt{{\mu }_{0}{\varepsilon }_{0}}{\bf{H}}$$

4 $${\bf{B}}={\mu }_{0}{\mu }_{r}{\bf{H}}-i\kappa \sqrt{{\mu }_{0}{\varepsilon }_{0}}{\bf{E}}$$

where *κ*, *ε*_*r*_ and *μ*_*r*_ denote, respectively, the chirality parameter, relative permittivity and permeability of the chiral medium.

The EM fields existing within the chiral coating (internal fields) can be represented by a combination of the spherical VWFs, in the following form^16^

5 $$\begin{array}{rcl}{{\bf{E}}}^{w} & = & {E}_{0}\,\sum _{m=-\infty }^{\infty }\,\sum _{n=|m|}^{\infty }\,\{{c}_{mn}[{{\bf{M}}}_{mn}^{(1)}({k}_{+})+{{\bf{N}}}_{mn}^{(1)}({k}_{+})]+{c}_{mn}^{^{\prime} }[{{\bf{M}}}_{mn}^{(3)}({k}_{+})+{{\bf{N}}}_{mn}^{(3)}({k}_{+})]\\  &  & +\,{d}_{mn}[{{\bf{M}}}_{mn}^{(1)}({k}_{-})-{{\bf{N}}}_{mn}^{(1)}({k}_{-})]+{d}_{mn}^{^{\prime} }[{{\bf{M}}}_{mn}^{(3)}({k}_{-})-{{\bf{N}}}_{mn}^{(3)}({k}_{-})]\}\end{array}$$

6 $$\begin{array}{rcl}{{\bf{H}}}^{w} & = & -\,i\frac{1}{\eta }{E}_{0}\,\sum _{m=-\infty }^{\infty }\,\sum _{n=|m|}^{\infty }\,\{{c}_{mn}[{{\bf{M}}}_{mn}^{(1)}({k}_{+})+{{\bf{N}}}_{mn}^{(1)}({k}_{+})]+{c}_{mn}^{^{\prime} }[{{\bf{M}}}_{pq}^{(3)}({k}_{+})+{{\bf{N}}}_{mn}^{(3)}({k}_{+})]\\  &  & -\,{d}_{mn}[{{\bf{M}}}_{mn}^{(1)}({k}_{-})-{{\bf{N}}}_{mn}^{(1)}({k}_{-})]-{d}_{mn}^{^{\prime} }[{{\bf{M}}}_{mn}^{(3)}({k}_{-})-{{\bf{N}}}_{mn}^{(3)}({k}_{-})]\}\end{array}$$

where $$\eta ={\eta }_{0}\sqrt{{\mu }_{r}/{\varepsilon }_{r}}$$, $${k}_{\pm }={k}_{0}(\sqrt{{\mu }_{r}{\varepsilon }_{r}}\pm \kappa )$$.

Eqs (1, 2, 5 and 6) are obtained in the MoM scheme, i.e., expanding the scattered and internal fields by using appropriate spherical VWFs as basis functions.

If the boundary conditions is writed, i.e., continuity of the tangential components of the EM fields at interface *S* between the chiral coating and free space

7 $$\hat{n}\times ({{\bf{E}}}^{s}+{{\bf{E}}}^{i})=\hat{n}\times {{\bf{E}}}^{w}$$

8 $$\hat{n}\times ({{\bf{H}}}^{s}+{{\bf{H}}}^{i})=\hat{n}\times {{\bf{H}}}^{w}$$

and vanishing of the tangential components of the electric field at *S*_1_ (inner conducting object’s surface)

9 $${\hat{n}}_{1}\times {{\bf{E}}}^{w}=0$$

In Eqs (7–9), **E**^*i*^ and **H**^*i*^ denote the incident electric and magnetic fields, and, $\hat{n}$ and ${\hat{n}}_{1}$ are respectively the outward unit normals to *S* and *S*_1_.

By virtue of Eqs (1, 2, 5 and 6), the boundary conditions in Eqs (7 and 8) are written as

10 $$\begin{array}{l}\hat{n}\times {E}_{0}\,\sum _{m=-\infty }^{\infty }\,\sum _{n=|m|}^{\infty }\,[{\alpha }_{mn}{{\bf{M}}}_{mn}^{(3)}({k}_{0})+{\beta }_{mn}{{\bf{N}}}_{mn}^{(3)}({k}_{0})]+\hat{n}\times {{\bf{E}}}^{i}\\ \begin{array}{rcl} & = & \hat{n}\times {E}_{0}\,\sum _{m=-\infty }^{\infty }\,\sum _{n=|m|}^{\infty }\,\{{c}_{mn}[{{\bf{M}}}_{mn}^{(1)}({k}_{+})+{{\bf{N}}}_{mn}^{(1)}({k}_{+})]+{c}_{mn}^{^{\prime} }[{{\bf{M}}}_{mn}^{(3)}({k}_{+})+{{\bf{N}}}_{mn}^{(3)}({k}_{+})]\\  &  & +\,{d}_{mn}[{{\bf{M}}}_{mn}^{(1)}({k}_{-})-{{\bf{N}}}_{mn}^{(1)}({k}_{-})]+{d}_{mn}^{^{\prime} }[{{\bf{M}}}_{mn}^{(3)}({k}_{-})-{{\bf{N}}}_{mn}^{(3)}({k}_{-})]\}\end{array}\end{array}$$

11 $$\begin{array}{l}\hat{n}\times {E}_{0}\,\sum _{m=-\infty }^{\infty }\,\sum _{n=|m|}^{\infty }\,[{\alpha }_{mn}{{\bf{N}}}_{mn}^{(3)}({k}_{0})+{\beta }_{mn}{{\bf{M}}}_{mn}^{(3)}({k}_{0})]+i{\eta }_{0}\hat{n}\times {{\bf{H}}}^{i}\\ \begin{array}{rcl} & = & \hat{n}\times \frac{{\eta }_{0}}{\eta }{E}_{0}\,\sum _{m=-\infty }^{\infty }\,\sum _{n=|m|}^{\infty }\,\{{c}_{mn}[{{\bf{M}}}_{mn}^{(1)}({k}_{+})+{{\bf{N}}}_{mn}^{(1)}({k}_{+})]+{c}_{mn}^{^{\prime} }[{{\bf{M}}}_{pq}^{(3)}({k}_{+})+{{\bf{N}}}_{mn}^{(3)}({k}_{+})]\\  &  & -\,{d}_{mn}[{{\bf{M}}}_{mn}^{(1)}({k}_{-})-{{\bf{N}}}_{mn}^{(1)}({k}_{-})]-{d}_{mn}^{^{\prime} }[{{\bf{M}}}_{mn}^{(3)}({k}_{-})-{{\bf{N}}}_{mn}^{(3)}({k}_{-})]\}\end{array}\end{array}$$

Eqs (10 and 11) are respectively multiplied (dot product) by the spherical VWFs ${{\bf{M}}}_{m^{\prime} n^{\prime} }^{(1)}({k}_{0})$ and ${{\bf{N}}}_{m^{\prime} n^{\prime} }^{(1)}({k}_{0})$, and then integrated over *S*, the following equations are obtained

12 $$\begin{array}{rcl}-\,{\oint }_{S}\,{{\bf{M}}}_{m^{\prime} n^{\prime} }^{r(1)}({k}_{0})\times {{\bf{E}}}^{i}\cdot \hat{n}dS & = & {E}_{0}\,\sum _{m=-\infty }^{\infty }\,\sum _{n=|m|}^{\infty }\,[{U}_{m^{\prime} n^{\prime} mn}{\alpha }_{mn}+{V}_{m^{\prime} n^{\prime} mn}{\beta }_{mn}]\\  &  & -\,{E}_{0}\,\sum _{m=-\infty }^{\infty }\,\sum _{n=|m|}^{\infty }\,\{[{U}_{m^{\prime} n^{\prime} mn}^{(1+)}+{V}_{m^{\prime} n^{\prime} mn}^{(1+)}]{c}_{mn}\\  &  & +\,[{U}_{m^{\prime} n^{\prime} mn}^{(3+)}+{V}_{m^{\prime} n^{\prime} mn}^{(3+)}]{c}_{mn}^{^{\prime} }\\  &  & +\,[{U}_{m^{\prime} n^{\prime} mn}^{(1-)}-{V}_{m^{\prime} n^{\prime} mn}^{(1-)}]{d}_{mn}\\  &  & +\,[{U}_{m^{\prime} n^{\prime} mn}^{(3-)}-{V}_{m^{\prime} n^{\prime} mn}^{(3-)}]{d}_{mn}^{^{\prime} }\}\end{array}$$

13 $$\begin{array}{rcl}-\,{\oint }_{S}\,{{\bf{N}}}_{m^{\prime} n^{\prime} }^{r(1)}({k}_{0})\times {{\bf{E}}}^{i}\cdot \hat{n}dS & = & {E}_{0}\,\sum _{m=-\infty }^{\infty }\,\sum _{n=|m|}^{\infty }\,[{K}_{m^{\prime} n^{\prime} mn}{\alpha }_{mn}+{L}_{m^{\prime} n^{\prime} mn}{\beta }_{mn}]\\  &  & -\,{E}_{0}\,\sum _{m=-\infty }^{\infty }\,\sum _{n=|m|}^{\infty }\,\{[{K}_{m^{\prime} n^{\prime} mn}^{(1+)}+{L}_{m^{\prime} n^{\prime} mn}^{(1+)}]{c}_{mn}\\  &  & +\,[{K}_{m^{\prime} n^{\prime} mn}^{(3+)}+{L}_{m^{\prime} n^{\prime} mn}^{(3+)}]{c}_{mn}^{^{\prime} }\\  &  & +\,[{K}_{m^{\prime} n^{\prime} mn}^{(1-)}-{L}_{m^{\prime} n^{\prime} mn}^{(1-)}]{d}_{mn}\\  &  & +\,[{K}_{m^{\prime} n^{\prime} mn}^{(3-)}-{L}_{m^{\prime} n^{\prime} mn}^{(3-)}]{d}_{mn}^{^{\prime} }\}\end{array}$$

14 $$\begin{array}{rcl}-\,i{\eta }_{0}\,{\oint }_{S}\,{{\bf{M}}}_{m^{\prime} n^{\prime} }^{r(1)}({k}_{0})\times {{\bf{H}}}^{i}\cdot \hat{n}dS & = & {E}_{0}\,\sum _{m=-\infty }^{\infty }\,\sum _{n=|m|}^{\infty }[{V}_{m^{\prime} n^{\prime} mn}{\alpha }_{mn}+{U}_{m^{\prime} n^{\prime} mn}{\beta }_{mn}]\\  &  & -\,\frac{{\eta }_{0}}{\eta }{E}_{0}\,\sum _{m=-\infty }^{\infty }\,\sum _{n=|m|}^{\infty }\,\{[{U}_{m^{\prime} n^{\prime} mn}^{(1+)}+{V}_{m^{\prime} n^{\prime} mn}^{(1+)}]{c}_{mn}\\  &  & +\,[{U}_{m^{\prime} n^{\prime} mn}^{(3+)}+{V}_{m^{\prime} n^{\prime} mn}^{(3+)}]{c}_{mn}^{^{\prime} }\\  &  & -\,[{U}_{m^{\prime} n^{\prime} mn}^{(1-)}-{V}_{m^{\prime} n^{\prime} mn}^{(1-)}]{d}_{mn}\\  &  & -\,[{U}_{m^{\prime} n^{\prime} mn}^{(3-)}-{V}_{m^{\prime} n^{\prime} mn}^{(3-)}]{d}_{mn}^{^{\prime} }\}\end{array}$$

15 $$\begin{array}{rcl}-\,i{\eta }_{0}\,{\oint }_{S}\,{{\bf{N}}}_{m^{\prime} n^{\prime} }^{r(1)}({k}_{0})\times {{\bf{H}}}^{i}\cdot \hat{n}^{\prime} dS^{\prime}  & = & {E}_{0}\,\sum _{m=-\infty }^{\infty }\,\sum _{n=|m|}^{\infty }\,[{L}_{m^{\prime} n^{\prime} mn}{\alpha }_{mn}+{K}_{m^{\prime} n^{\prime} mn}{\beta }_{mn}]\\  &  & -\,\frac{{\eta }_{0}}{\eta }{E}_{0}\,\sum _{m=-\infty }^{\infty }\,\sum _{n=|m|}^{\infty }\,\{[{K}_{m^{\prime} n^{\prime} mn}^{(1+)}+{L}_{m^{\prime} n^{\prime} mn}^{(1+)}]{c}_{mn}\\  &  & +\,[{K}_{m^{\prime} n^{\prime} mn}^{(3+)}+{L}_{m^{\prime} n^{\prime} mn}^{(3+)}]{c}_{mn}^{^{\prime} }\\  &  & -\,[{K}_{m^{\prime} n^{\prime} mn}^{(1-)}-{L}_{m^{\prime} n^{\prime} mn}^{(1-)}]{d}_{mn}\\  &  & -\,[{K}_{m^{\prime} n^{\prime} mn}^{(3-)}-{L}_{m^{\prime} n^{\prime} mn}^{(3-)}]{d}_{mn}^{^{\prime} }\}\end{array}$$

The explicit expressions of *U*_*m*′*n*′*mn*_, *V*_*m*′*n*′*mn*_, *K*_*m*′*n*′*mn*_ and *L*_*m*′*n*′*mn*_ are given by

16 $${U}_{m^{\prime} n^{\prime} mn}={\oint }_{S}\,{{\bf{M}}}_{m^{\prime} n^{\prime} }^{(1)}({k}_{0})\times {{\bf{M}}}_{mn}^{(3)}({k}_{0})\cdot \hat{n}dS$$

17 $${V}_{m^{\prime} n^{\prime} mn}={\oint }_{S}\,{{\bf{M}}}_{m^{\prime} n^{\prime} }^{(1)}({k}_{0})\times {{\bf{N}}}_{mn}^{(3)}({k}_{0})\cdot \hat{n}dS$$

18 $${K}_{m^{\prime} n^{\prime} mn}={\oint }_{S}\,{{\bf{N}}}_{m^{\prime} n^{\prime} }^{(1)}({k}_{0})\times {{\bf{M}}}_{mn}^{(3)}({k}_{0})\cdot \hat{n}dS$$

19 $${L}_{m^{\prime} n^{\prime} mn}={\oint }_{S}\,{{\bf{N}}}_{m^{\prime} n^{\prime} }^{(1)}({k}_{0})\times {{\bf{N}}}_{mn}^{(3)}({k}_{0})\cdot \hat{n}dS$$

and those of ${U}_{m^{\prime} n^{\prime} mn}^{(j\pm )}$, ${V}_{m^{\prime} n^{\prime} mn}^{(j\pm )}$, ${K}_{m^{\prime} n^{\prime} mn}^{(j\pm )}$ and ${L}_{m^{\prime} n^{\prime} mn}^{(j\pm )}$ are determined by replacing respectively ${{\bf{M}}}_{mn}^{(3)}({k}_{0})$ or ${{\bf{N}}}_{mn}^{(3)}({k}_{0})$ in *U*_*m*′*n*′*mn*_, *V*_*m*′*n*′*mn*_, *K*_*m*′*n*′*mn*_ and *L*_*m*′*n*′*mn*_ with ${{\bf{M}}}_{mn}^{(j)}({k}_{\pm })$ or ${{\bf{N}}}_{mn}^{(j)}({k}_{\pm })$ (*j* = 1, 3).

Eqs (12–15) are interpreted as follows. The scattered and internal fields are excited due to the incident fields **E**^*i*^ and **H**^*i*^. So, the incident EM beam can be considered as a “source”, and the scattered and internal fields as the subsequent “responses”. The spherical VWFs ${{\bf{M}}}_{m^{\prime} n^{\prime} }^{(1)}({k}_{0})$ and ${{\bf{N}}}_{m^{\prime} n^{\prime} }^{(1)}({k}_{0})$ are usually used to expand an incident EM beam, and then they are chosen as the weighting functions to derive Eqs (12–15) following the MoM procedure.

A substitution of Eqs (5 and 6) into Eq. (9) leads to

20 $$\begin{array}{l}{\hat{n}}_{1}\times \sum _{m=-\infty }^{\infty }\,\sum _{n=|m|}^{\infty }\,\{{c}_{mn}[{{\bf{M}}}_{mn}^{(1)}({k}_{+})+{{\bf{N}}}_{mn}^{(1)}({k}_{+})]+{c}_{mn}^{^{\prime} }[{{\bf{M}}}_{mn}^{(3)}({k}_{+})+{{\bf{N}}}_{mn}^{(3)}({k}_{+})]\\ \,+\,{d}_{mn}[{{\bf{M}}}_{mn}^{(1)}({k}_{-})-{{\bf{N}}}_{mn}^{(1)}({k}_{-})]+{d}_{mn}^{^{\prime} }[{{\bf{M}}}_{mn}^{(3)}({k}_{-})-{{\bf{N}}}_{mn}^{(3)}({k}_{-})]\}=0\end{array}$$

The combinations of the spherical VWFs ${{\bf{M}}}_{mn}^{(j)}({k}_{+})+{{\bf{N}}}_{mn}^{(j)}({k}_{+})$ and ${{\bf{M}}}_{mn}^{(j)}({k}_{-})-{{\bf{N}}}_{mn}^{(j)}({k}_{-})$ (*j* = 1, 3) in Eq. (20) describe two eigenwaves (right and left-handed Beltrami waves) within the chiral coating^8^, and they also respectively represent the Beltrami waves propagating towards or scattered from the inner conducting object when the superscript *j* = 1 or 3. Motivated by the derivation of Eqs (12–15), we have Eq. (20) multiplied (dot product) by the weighting functions ${{\bf{M}}}_{m^{\prime} n^{\prime} }^{(1)}({k}_{+})+{{\bf{N}}}_{m^{\prime} n^{\prime} }^{(1)}({k}_{+})$ and ${{\bf{M}}}_{m^{\prime} n^{\prime} }^{(1)}({k}_{-})-{{\bf{N}}}_{m^{\prime} n^{\prime} }^{(1)}({k}_{-})$ respectively, and then integrated over *S*_1_. After performing the above mathematical operations, we can readily obtain

21 $$\begin{array}{l}\sum _{m=-\infty }^{\infty }\,\sum _{n=|m|}^{\infty }\,\{[{U}_{m^{\prime} n^{\prime} mn}^{(1+,1+)}+{V}_{m^{\prime} n^{\prime} mn}^{(1+,1+)}+{K}_{m^{\prime} n^{\prime} mn}^{(1+,1+)}+{L}_{m^{\prime} n^{\prime} mn}^{(1+,1+)}]{c}_{mn}\\ \,+\,[{U}_{m^{\prime} n^{\prime} mn}^{(1+,3+)}+{V}_{m^{\prime} n^{\prime} mn}^{(1+,3+)}+{K}_{m^{\prime} n^{\prime} mn}^{(1+,3+)}+{L}_{m^{\prime} n^{\prime} mn}^{(1+,3+)}]{c}_{mn}^{^{\prime} }\\ \,+\,[{U}_{m^{\prime} n^{\prime} mn}^{(1+,1-)}-{V}_{m^{\prime} n^{\prime} mn}^{(1+,1-)}+{K}_{m^{\prime} n^{\prime} mn}^{(1+,1-)}-{L}_{m^{\prime} n^{\prime} mn}^{(1+,1-)}]{d}_{mn}\\ \,+\,[{U}_{m^{\prime} n^{\prime} mn}^{(1+,3-)}-{V}_{m^{\prime} n^{\prime} mn}^{(1+,3-)}+{K}_{m^{\prime} n^{\prime} mn}^{(1+,3-)}-{L}_{m^{\prime} n^{\prime} mn}^{(1+,3-)}]{d^{\prime} }_{mn}\}=0\end{array}$$

22 $$\begin{array}{l}\sum _{m=-\infty }^{\infty }\,\sum _{n=|m|}^{\infty }\,\{[{U}_{m^{\prime} n^{\prime} mn}^{(1-,1+)}+{V}_{m^{\prime} n^{\prime} mn}^{(1-,1+)}-{K}_{m^{\prime} n^{\prime} mn}^{(1-,1+)}-{L}_{m^{\prime} n^{\prime} mn}^{(1-,1+)}]{c}_{mn}\\ \,+\,[{U}_{m^{\prime} n^{\prime} mn}^{(1-,3+)}+{V}_{m^{\prime} n^{\prime} mn}^{(1-,3+)}-{K}_{m^{\prime} n^{\prime} mn}^{(1-,3+)}+{L}_{m^{\prime} n^{\prime} mn}^{(1-,3+)}]{c}_{mn}^{^{\prime} }\\ \,+\,[{U}_{m^{\prime} n^{\prime} mn}^{(1-,1-)}-{V}_{m^{\prime} n^{\prime} mn}^{(1-,1-)}-{K}_{m^{\prime} n^{\prime} mn}^{(1-,1-)}+{L}_{m^{\prime} n^{\prime} mn}^{(1-,1-)}]{d}_{mn}\\ \,+\,[{U}_{m^{\prime} n^{\prime} mn}^{(1-,3-)}-{V}_{m^{\prime} n^{\prime} mn}^{(1-,3-)}-{K}_{m^{\prime} n^{\prime} mn}^{(1-,3-)}+{L}_{m^{\prime} n^{\prime} mn}^{(1-,3-)}]{d}_{mn}^{^{\prime} }\}=0\end{array}$$

where (*j* = 1, 3)

23 $${U}_{m^{\prime} n^{\prime} mn}^{(1\pm ,j\pm )}={\oint }_{{S}_{1}}\,{{\bf{M}}}_{m^{\prime} n^{\prime} }^{(1)}({k}_{\pm })\times {{\bf{M}}}_{mn}^{(j)}({k}_{\pm })\cdot {\hat{n}}_{1}d{S}_{1}$$

24 $${V}_{m^{\prime} n^{\prime} mn}^{(1\pm ,j\pm )}={\oint }_{{S}_{1}}\,{{\bf{M}}}_{m^{\prime} n^{\prime} }^{(1)}({k}_{\pm })\times {{\bf{N}}}_{mn}^{(j)}({k}_{\pm })\cdot {\hat{n}}_{1}d{S}_{1}$$

25 $${K}_{m^{\prime} n^{\prime} mn}^{(1\pm ,j\pm )}={\oint }_{{S}_{1}}\,{{\bf{N}}}_{m^{\prime} n^{\prime} }^{(1)}({k}_{\pm })\times {{\bf{M}}}_{mn}^{(j)}({k}_{\pm })\cdot {\hat{n}}_{1}d{S}_{1}$$

26 $${L}_{m^{\prime} n^{\prime} mn}^{(1\pm ,j\pm )}={\oint }_{{S}_{1}}\,{{\bf{N}}}_{m^{\prime} n^{\prime} }^{(1)}({k}_{\pm })\times {{\bf{N}}}_{mn}^{(j)}({k}_{\pm })\cdot {\hat{n}}_{1}d{S}_{1}$$

Eqs (12–15 and 21 and 22) provide an infinite system of linear equations to determine the expansion coefficients *α*_*mn*_, *β*_*mn*_, *c*_*mn*_, ${c}_{mn}^{^{\prime} }$, *d*_*mn*_ and ${d}_{mn}^{^{\prime} }$, as the explicit expressions of **E**^*i*^ and **H**^*i*^ are known.

Since the beam description of EM field components is usually obtained in its own system *O*′*x*′*y*′*z*′, to evaluate numerically the surface integrals including **E**^*i*^ and **H**^*i*^ in Eqs (12–15) the following transformations ought to be carried out from *O*′*x*′*y*′*z*′ to the scatterer system *Oxyz*

27 $$(\begin{array}{c}x^{\prime} -{x}_{0}\\ y^{\prime} -{y}_{0}\\ z^{\prime} -{z}_{0}\end{array})=T(\begin{array}{c}x\\ y\\ z\end{array}),\,(\begin{array}{c}{A}_{x^{\prime} }\\ {A}_{y^{\prime} }\\ {A}_{z^{\prime} }\end{array})=T(\begin{array}{c}{A}_{x}\\ {A}_{y}\\ {A}_{z}\end{array})$$

where *A* is described the electric or magnetic field, and the transformation matrix *T* is computed by

28 $$T=(\begin{array}{ccc}\cos \,\beta  & 0 & -\,\sin \,\beta \\ 0 & 1 & 0\\ \sin \,\beta  & 0 & \cos \,\beta \end{array})\,(\begin{array}{ccc}\cos \,\alpha  & \sin \,\alpha  & 0\\ -\,\sin \,\alpha  & \cos \,\alpha  & 0\\ 0 & 0 & 1\end{array})$$

We apply Simpson’s 1/3 rule to evaluate numerically the surface integrals in Eqs (12–15 and 23–26), where the expressions of both $\hat{n}dS$ and ${\hat{n}}_{1}d{S}_{1}$ follow^17^. In order to solve the infinite system consisting of Eqs (12–15 and 21 and 22) for the unknown expansion coefficients, it can be truncated the series by setting *n* = |*m*|, |*m*| + 1, …, |*m*| + *N* and *n*′ = |*m*′|, |*m*′| + 1, …, |*m*′| + *N*, given each of *m* = −*M*, −*M* + 1, …, *M* and *m*′ = −*M*, −*M* + 1, …, *M*. In our computations, *M* and *N* (usually larger than 8 and 20 respectively) are so chosen to ensure a solution accuracy better than three or more significant figures, and the Gaussian eliminated technique is utilized in the MATLAB environment for solving these 6(2*M* + 1) (*N* + 1) unknowns. When both the chiral coating and inner conducting object have the *z* axis as a rotation axis (axisymmetric object), the different *m* indices will decouple since the surface integrals in Eqs (16–19 and 23–26) are zero when *m* ≠ −*m*′^9,17^. Then, Eqs (12–15 and 21 and 22) become a 6(*N* + 1) matrix equation for each of *m* = −*m*′ = −*M*, −*M* + 1, …, *M*.

Generally, compared with the usual MoM solution such as in^11^, the advantages of the above MoM based semi-analytical theoretical procedure are obvious. Instead of using the triangular rooftop vector functions, the corresponding spherical VWFs are adopted as the basis and weighting functions, so most of the formulations are described by analytical expressions. As a result, the number of unknowns that have to be determined is greatly reduced, especially for an axisymmetric object, and then a significant saving of computer time and memory can be achieved to solve for them. Moreover, the current MoM scheme is directly applied to the boundary conditions rather than to the combined field integral equations based on the surface equivalence principle, which is simple in theory and also easy to manipulate mathematically.

## Numerical Results

In this section, we will focus on the far-zone scattered field which is often of practical significance. By using the asymptotic form of **E**^*s*^ as *k*_0_*r* → ∞, the differential scattering cross section (DSCS) is defined in^8,9^

29 $$\sigma (\theta ,\varphi )=4\pi {r}^{2}{|\frac{{{\boldsymbol{{\rm E}}}}^{s}}{{E}_{0}}|}^{2}=\frac{{\lambda }^{2}}{\pi }(|{T}_{1}(\theta ,\varphi ){|}^{2}+|{T}_{2}(\theta ,\varphi ){|}^{2})$$

where

30 $${T}_{1}(\theta ,\varphi )=\sum _{m=-\infty }^{\infty }\,\sum _{n=|m|}^{\infty }{(-i)}^{n}\,[m\frac{{P}_{n}^{m}(\cos \,\theta )}{\sin \,\theta }{\alpha }_{mn}+\frac{d{P}_{n}^{m}(\cos \,\theta )}{d\theta }{\beta }_{mn}]$$

31 $${T}_{2}(\theta ,\varphi )=\sum _{m=-\infty }^{\infty }\,\sum _{n=|m|}^{\infty }\,{(-i)}^{n-1}\,[\frac{d{P}_{n}^{m}(\cos \,\theta )}{d\theta }{\alpha }_{mn}+m\frac{{P}_{n}^{m}(\cos \,\theta )}{\sin \,\theta }{\beta }_{mn}]$$

In the following calculations, we are restricted to the Gaussian beam, CPW, ZOBB and HED radiated field illuminating from a chiral-coated conducting spheroid and finite-length circular cylinder, i.e., a conducting spheroid coated with a chiral spheroid layer (semimajor and semiminor axes denoted by *a* and *b* for the spheroid coating, and by *a*_1_ and *b*_1_ for the inner conducting spheroid) and a conducting cylinder coated with a chiral cylinder layer (length and cross section radius denoted by 2*l*_0_ and *r*_0_ for the cylinder coating, and by 2*l*_1_ and *r*_1_ for the inner conducting cylinder).

**Figure 2:**
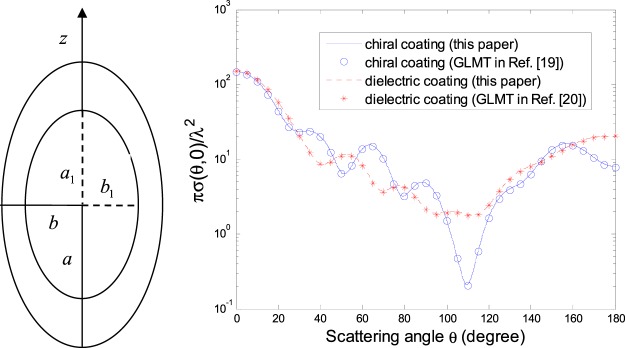
Normalized DSCS *πσ*(*θ*, 0)/*λ*^2^ for a chiral-coated conducting spheroid (*k*_0_*a*_1_ = 6, *a*_1_/*b*_1_ = 2, *k*_0_*a* = 9.14, *a*/*b* = 2, *ε*_*r*_ = 4, *μ*_*r*_ = 1, *κ* = 0.5) and that for a dielectric-coated conducting spheroid (similarly as the former but *κ* = 0), both illuminated by a Gaussian beam (TE mode, *w*_0_ = 2*λ*, *α* = *β* = 0, *x*_0_ = *y*_0_ = *z*_0_ = 0).

Figure 2 is shown that the normalized DSCS *πσ*(*θ*, 0)/*λ*^2^ for a conducting spheroid either with a chiral or dielectric spheroid coating, illuminated by a Gaussian beam (TE mode) following the Davis first-order expression^18^. In Fig. 2, the numerical results calculated by the present solution are also compared with those by the generalized Lorenz–Mie theory (GLMT) that gives an exact analytical procedure for a coated conducting spheroid in^19^ and^20^. As expected, excellent agreements are observed in Fig. 2, which to a certain extent validates the proposed method.

**Figure 3:**
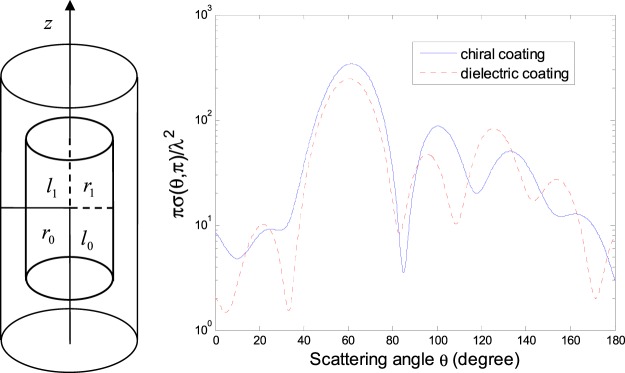
Normalized DSCS *πσ*(*θ*, *π*)/*λ*^2^ for a chiral-coated conducting cylinder (*k*_0_*l*_1_ = *π*, *l*_1_/*r*_1_ = 1, *k*_0_*l*_0_ = 1.5*π*, *l*_0_/*r*_0_ = 1, *ε*_*r*_ = 4, *μ*_*r*_ = 1, *κ* = 0.5) and that for a dielectric-coated conducting cylinder (similarly as the former but *κ* = 0), both illuminated by a Gaussian beam (TE mode, *w*_0_ = 5*λ*, *α* = 0, $\beta =\tfrac{\pi }{3}$, *x*_0_ = *y*_0_ = *z*_0_ = 0).

The normalized DSCSs *πσ*(*θ*, *π*)/*λ*^2^ are shown in Fig. 3 for a conducting circular cylinder coated either with a chiral or dielectric cylinder layer, under Gaussian beam illumination as in plotting Fig. 2.

From Figs 2 and 3 we can see that the conducting spheroid and finite-length circular cylinder, whether with a chiral or dielectric coating, have the maximum DSCS around *θ* = *β*, i.e., the maximum forward scattering. In addition, compared with the case of a dielectric coating, the difference in the normalized DSCS is obvious for a chiral-coated layer.

**Figure 4:**
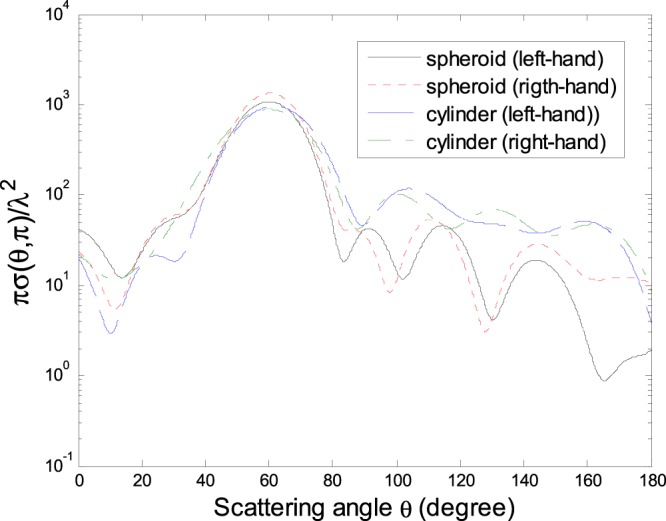
Normalized DSCSs *πσ*(*θ*, *π*)/*λ*^2^ for a chiral-coated conducting spheroid (spheroid) and a chiral-coated conducting finite-length circular cylinder (cylinder) respectively as in Figs 2 and 3, but illuminated by the left-hand (left-hand) and right-hand (right-hand) CPWs (*α* = 0, $\beta =\tfrac{\pi }{3}$, *x*_0_ = *y*_0_ = *z*_0_ = 0).

It is well-known that the left- and right-hand CPWs (electric and magnetic fields described as ${{\bf{E}}}^{i}={E}_{0}(\hat{x^{\prime} }\pm i\hat{y}^{\prime} ){e}^{i{k}_{0}z^{\prime} }$,${{\bf{H}}}^{i}=\frac{{E}_{0}}{{\eta }_{0}}(\,\mp \,i\hat{x}^{\prime} +\hat{y}^{\prime} ){e}^{i{k}_{0}z^{\prime} }$ in *O*′*x*′*y*′*z*′) are different in their action on chiral media^21^. The difference in the normalized DSCS *πσ*(*θ*, *π*)/*λ*^2^ is shown in Fig. 4 for a chiral-coated conducting spheroid and finite-length circular cylinder as in Figs 2 and 3.

**Figure 5:**
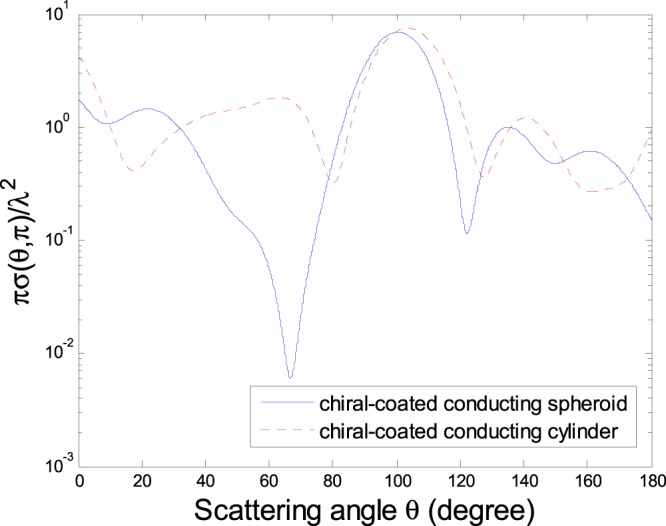
Normalized DSCSs *πσ*(*θ*, *π*)/*λ*^2^ of a chiral-coated conducting spheroid and a chiral-coated conducting finite-length circular cylinder respectively as in Figs 2 and 3, under the illumination of a ZOBB (*α* = 0, $\beta =\tfrac{\pi }{4}$, half-cone angle $\zeta =\tfrac{\pi }{3}$, *x*_0_ = *y*_0_ = *z*_0_ = 0).

As a diffraction free beam, the ZOBB has gained growing attention in various fields^22–24^. A detailed description of the ZOBB propagating along the positive *z*′ axis in *O*′*x*′*y*′*z*′ has been given^23,24^. Figure 5 is shown the normalized DSCS *πσ*(*θ*, *π*)/*λ*^2^ of a chiral-coated conducting spheroid and finite-length circular cylinder as in Figs 2 and 3 but under the illumination of a ZOBB. From Fig. 5 we can find that, as opposed to the case of a Gaussian beam, the maximum forward scattering dose not appear in the numerical results. In addition, the maximum DSCS for a Gaussian beam is usually larger than that for a ZOBB.

The EM fields radiated from a HED oriented along the *z*′ axis and located at origin *O*′ are expressed as^25^:

32 $${{\bf{E}}}^{i}({\bf{r}}^{\prime} )={E}_{0}[\hat{z}^{\prime} +\frac{1}{{k}_{0}^{2}}\nabla ^{\prime} \frac{\partial }{\partial z^{\prime} }]\frac{{e}^{i{k}_{0}r^{\prime} }}{{k}_{0}r^{\prime} }$$

33 $${{\bf{H}}}^{i}({\bf{r}}^{\prime} )=-\,i\frac{{E}_{0}}{{\eta }_{0}}\frac{1}{{k}_{0}}\nabla ^{\prime} \frac{{e}^{i{k}_{0}r^{\prime} }}{{k}_{0}r^{\prime} }\times \hat{z}^{\prime} $$

where $r^{\prime} =\sqrt{{x^{\prime} }^{2}+{y^{\prime} }^{2}+{z^{\prime} }^{2}}$, ${E}_{0}=i\omega \mu Il\frac{{k}_{0}}{4\pi }$, and the prime denotes that the EM field expressions are written in *O*′*x*′*y*′*z*′.

**Figure 6:**
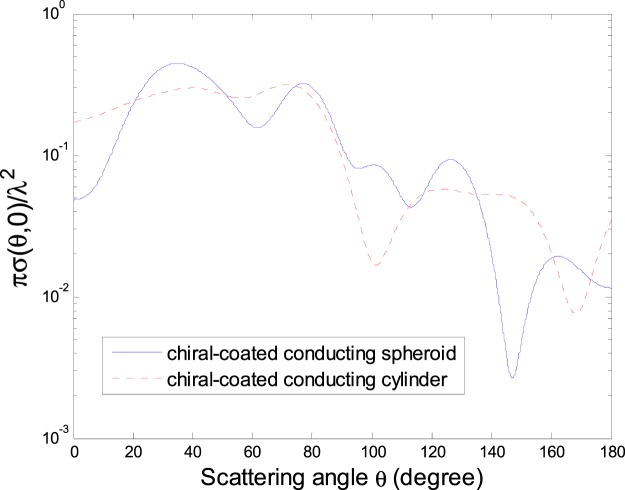
Normalized DSCS *πσ*(*θ*, 0)/*λ*^2^ for a chiral-coated conducting spheroid and that for a chiral-coated conducting circular cylinder respectively as in Figs 2 and 3, under the illumination of the HED radiation (*x*_0_ = 2*λ*, *y*_0_ = *z*_0_ = 0, *α* = *π*/6, *β* = *π*/4).

The normalized DSCS *πσ*(*θ*, 0)/*λ*^2^ is shown in Fig. 6 for the same models as in Figs 2 and 3 under the illumination of the HED radiated field.

## Conclusion

Based on a combination of the EM field expansions in infinite series of the spherical VWFs with the MoM scheme, a semi-analytical solution of the EM beam scattering from a chiral-coated conducting object is proposed. By taking as examples an incident Gaussian beam, VPW, ZOBB and HED radiation striking a chiral-coated conducting spheroid and finite-length circular cylinder, the normalized DSCS is calculated. The correctness of the present theory to a certain extent is validated by comparing the normalized DSCS for a conducting spheroid, either with a chiral or dielectric coating, illuminated by a Gaussian beam with those obtained by the GLMT that gives an exact analytical solution. Theoretically, the present MoM based scheme can be used to treat arbitrary EM beam scattering given their explicit expressions, even extended to an infinite cylinder when appropriate cylindrical VWFs are chosen as the basis and weighting functions.
